# Towards a methodology for testing models as hypotheses in the inexact sciences

**DOI:** 10.1098/rspa.2018.0862

**Published:** 2019-04-24

**Authors:** Keith Beven

**Affiliations:** Lancaster Environment Centre, Lancaster University, Lancaster, LA1 4YQ, UK

**Keywords:** epistemic uncertainty, runoff coefficients, hydrological models

## Abstract

This paper reviews the issues involved in treating hydrology as an example of an inexact science faced with significant epistemic uncertainties. It proposes a novel method for developing limits of acceptability for testing hydrological models as hypotheses about how a catchment hydrological system might function. The approach is based only on an analysis of the available observations and the consideration of event mass balance for successive rainfall-runoff events. It is shown that there are many events that are subject to epistemic uncertainties in the input data so that mass balance is not satisfied. The proposed approach allows taking these epistemic uncertainties into account in a pragmatic way before any model runs are made. It is an approach that might be applicable in other areas of environmental science where similar basic principles are fundamental to models, but which might not be satisfied by the observations that are used for model evaluation.

## 1. Introduction

Some 15 years ago, I wrote an article for this journal ‘Towards a coherent philosophy for environmental modelling’ [1] that had its basis in my experience as a hydrologist and trying to do hydrological science in the face of epistemic uncertainties (uncertainties resulting from lack of knowledge) in both hydrological data and the representation of hydrological processes. This has been a continuing theme in my research (see also [2–10]). I made the point in that article that most practicing environmental modellers have a ‘pragmatic realist’ philosophy in which they like to think that the variables in their computer programmes represent real masses and fluxes in the environment while at the same time recognizing the approximate nature of their science and its observable quantities. While philosophically such a positivist view may not really be tenable, it represents how many modellers actually work.

Environmental models, such as those in hydrology, are based in the physical and chemical sciences, but are necessarily associated with significant knowledge uncertainties because of the limitations in knowing how to close the system, how to specify the boundary conditions, how to properly represent the relevant processes at the scales of interest, how to define effective model parameter values and how to evaluate the model outputs against the observational data that are available. While all sciences are subject to more or less uncertainties, with the exception of the logical deductions of mathematics, as examples of where the uncertainties can be large, and controlled experiments at scales of interest can be difficult or impossible, we might refer to the environmental sciences as examples of the inexact sciences.

Hydrology is one of these inexact sciences that have been my subject of study for the last 40 years. When I started my research, I really wanted to understand the development of landforms but quickly realized that to do so required first getting the water flow processes right. The domain of the hydrologist starts when water reaches the vegetation or ground surface as precipitation in the form of rain or snow and ends when that water is lost from a particular catchment area as either stream discharge (when it becomes an input boundary condition for the oceanographer) or evapotranspiration back to the atmosphere (when it becomes an input boundary condition for the meteorologist). The scales of interest range from the movement of water in individual pores in the soil, to experimental plots, fields and hillslope scales, right up to the largest river catchment areas in the world. Water is also an important driver for the movement of nutrients, sediments and pollutants, and serves as an ecological habitat in soils, aquifers, rivers and lakes, but biological and chemical processes can also interact to have feedback effects on the water movement.

One of the fascinating things about hydrology is how to span those scales of interest, particularly when so much of the hydrological system, and particularly the subsurface, can only be poorly observed [2–4,11]. Doing so is not just a problem of physics and chemistry, of course, because as we start to consider larger scales, the impact of man in affecting water flows becomes more and more important. Since water is necessary for life and essential to so much of man's activities in the world, hydrological science has also been driven by the need for good water resources management. For millennia, man has organized irrigation systems, used water harvesting and redistribution using quanats, canals, aqueducts and pipes, extracted groundwaters, designed dams and drainage system, and devised schemes for dealing with waste waters (even if often a strategy of dilute and forget). Agriculture, forestry and urbanization has also modified the natural flow pathways in nearly all parts of the globe. It is clear that not all of these practices are managed sustainably, resulting in water management problems as a result of over abstractions from lakes, rivers and groundwater, and the pollution of water resources, in many parts of the world. Understanding these interactions is the aim of the current International Association of Hydrological Sciences decadal programme on sociohydrology called Panta Rhei (e.g. [12–14]; http://iahs.info/Commissions--W-Groups/Working-Groups/Panta-Rhei.do where the aims and science questions being addressed can be found).

This paper will not address all those questions. Its focus is on the evaluation of models of hydrological processes using available hydrological observations. There is a need to test such models against observations, in order to increase confidence in making predictions into the future to represent changes, either in boundary conditions (e.g. due to climate change) or the nature of the system as reflected in the model parameters (e.g. due to urban development, groundwater abstractions or afforestation of large areas). We might use different data-based methods for short-term forecasting or infilling of past data when it might not be so important to have a representation of the hydrological processes. Thus, we want the models to be based on good hydrological science, to be ‘right for the right reasons' [15]. The question is how to test being right for the right reasons or fit for purpose when both models and observations are subject to epistemic uncertainties. Model testing in this sense can be a form of abductive reasoning (in the sense of trying to find a parsimonious representation matching the available observations, e.g. [16]) even if model outputs are usually a deductive consequence of the input data and model structure. Predicting the future, and future change, remains a form of induction, subject to potential surprise resulting from lack of knowledge about future change, processes and boundary conditions (e.g. [17]). In this respect, therefore, we also seek methodologies robust to such uncertainties.

## 2. Why hydrology is a good example of an inexact science

The distinction between the exact and inexact sciences was in fact first made in respect of the social sciences (e.g. [18]) but we can no longer pretend that all the physical and chemical sciences are ‘exact’ in the sense of making predictions with very high signal-to-noise ratios, however, firm the theoretical foundations on which they are based. There are many reasons for this but they are mostly the result of sources of epistemic uncertainty. In hydrology, it is easy to tabulate a number of sources of epistemic uncertainty associated with system closure, boundary conditions, process representations and parametrizations and observables (e.g. [9,17]). Not all of these uncertainties are easily quantified (precisely *because* they result from lack of knowledge) and in most cases there is no prospect of finding new measurement techniques or using randomized experimental designs that will significantly reduce the degree of uncertainty and lead to greatly improved understanding. Thus, hydrology is necessarily an inexact science, with uncertainties that impact on the predictability of hydrological models for informing management decisions. In this, it has much in common with many other areas of the environmental sciences.

We can illustrate this by going back to the very earliest days of scientific hydrology. In the seventeenth century, there had still been some debate about whether rainfall and dewfall was sufficient to maintain the flows of springs and rivers. Perrault [19] and Marriotte [20] in France, working on parts of the Seine basin had suggested that this was the case. Edmund Halley (1656–1742), however, in a paper presented to the Royal Society in 1691, could not agree, arguing that subterranean condensation of rising vapours of water was necessary to account for the volumes of both river flow and evapotranspiration over land and sea surfaces [21–23]. Halley played a critical role in the recognition of evapotranspiration in the water balance of an area [24] and made some of the first observations of evaporation, notably at Gresham College in Oxford [25].

The question was taken up, a long time later, by John Dalton (1766–1844) in a paper presented to the Manchester Literary and Philosophical Society in 1799 and entitled: ‘Experiments and Observations to determine whether a Quantity of Rain and Dew is equal to the Quantity of Water carried off by the Rivers and raised by Evaporation; with an Enquiry into the origin of Springs.’ The paper was later published in the Memoirs of the Society [26]. In that paper Dalton made the first attempt to calculate a water balance for the whole of England and Wales, using the limited data on rainfalls measured at 30 sites, river flows (using Halley's estimates of flow in the River Thames) and his own estimates of evaporation rates. His water balance calculation suggested an average annual rainfall input of 31 inches, an additional input of 5 inches from dewfall, an average annual river flow of 13 inches and an average evaporation rate (allowing for observations on both water and grass surfaces) of 30 inches. As might be expected, in working at such a large scale and with such limited data, this does not give a closed water balance: there is a deficit of 7 inches. Rodda [27] revisited the calculations and suggested a lower figure for the evapotranspiration term, which would have reduced the deficit, but this is a good example of where limited knowledge meant that even the most basic equation in hydrology, that of mass balance, cannot be proven without allowing for significant uncertainty. Even with more-refined observational methods and data available today that remains the case, particularly for individual events (see [28], for examples in the Tyne catchment and also [29], for a more general discussion of the use of such constitutive laws).

That is not to say that progress cannot be made, but this has often depended on a new type of measurement becoming more widely available. Interestingly, the more widespread availability of remote sensing products, including estimation of rainfall by radar, has not yet had a dramatic impact in the representation of hydrological processes precisely *because* of the significant uncertainty associated with the interpretation of such products (albeit that it is widely used in short-term forecasting applications as a source of data for adaptive forecasting). It has perhaps had more impact in terms of thinking in terms of spatial structure in catchments, particularly in making available high resolution and relatively precise digital terrain data.

The most significant development in the last 50 years, in assessing whether model representations are based on the right reasoning, has probably been the more widespread use of tracers, especially the environmental isotopes of Deuterium and Oxygen 18 and atomic bomb testing derived tritium. The seasonal nature of isotopic concentrations in precipitation in many parts of the world, and the variability from event to event, have made it possible to study the transit time distributions of water in hillslope and catchment systems in ways not previously possible. Starting with the groundwater and precipitation studies of Eriksson [30,31], the later snowmelt work of Dincer *et al*. [32] and the overland flow and catchment discharge work of Sklash & Farvolden [33], environmental tracers revealed that the storm hydrograph from a catchment was often comprised predominantly of water stored in the catchment before a rainfall or snowmelt event and displaced from storage by the event inputs. This was a fundamental change to hydrological understanding which previously had generally assigned fast storm runoff to the overland flow of storm event water (even if there are papers going back to the 1930s that argue that this could not always be the case, e.g. [34]; see also [35–37]). Initially, such environmental isotope measurements were expensive, but further insights are now being gained as the measurement technology has developed to allow fine time resolution series to be observed (e.g. [38–40]).

## 3. The role of models in the inexact environmental sciences

There are two primary roles for models in the inexact sciences. The first is to provide a means of quantitatively testing the consequences of various formal hypotheses about the representation of processes in the system. This might be called exploratory (deductive or abductive) modelling for doing the science (e.g. [16]). The second is to provide (inductive) quantitative forecasts and simulations of future hydrological scenarios that might be used to inform decisions in water and land management, particularly about the impacts of change into the future. This might be called modelling for applying the science. Ideally of course, a satisfactory outcome of doing the science is achieved before it is applied in practice, but very often this has not been the case in the past because of data and model limitations. The science might be too difficult or the decisions too pressing to wait for a fully acceptable model of the processes to be employed (there are many examples of this from local to global scale modelling). It might even be the case that a model that is not really fit-for-purpose is used in decision making, either because of historical legacy within a particular paradigm or institutional framework, or because satisfactory representations of all the processes that are perceived as influencing the system are not yet available (e.g. [8,15,41]). To paraphrase George Box, we know only too well that all models are wrong but testing which models might be useful in the inexact sciences is still a difficult problem.

## 4. Model testing in the face of epistemic uncertainties

It is also a controversial problem. A decade or so ago, a number of hydrologists suggested that focusing too much attention on the uncertainties in the modelling process would result in undermining the science and the perception of the utility of that science by users (see, for example, the discussion on undermining the science summarized in [42]). Similar concerns are regularly aired in other areas of environmental modelling, including climate models where uncertainties have been seized upon as a reason for inaction. Different opinions on testing hydrological models as hypotheses have been expressed in opinion pieces (for example, the differing views of [43]; and [44]) and the series of recent ‘debates’ papers [8,16,45]. Different views and philosophies on how to treat uncertainties have also been discussed by Beven [7] and Nearing *et al*. [46]. This remains a controversial topic that is unlikely to be resolved quickly, again precisely *because* of the epistemic nature of the uncertainties.

So let us consider some of the issues that arise in testing process models in the hydrology and other environmental science. What follows will focus on process-based models that represent attempts to incorporate scientific knowledge into the modelling process. There are other ways of making predictions of system behaviour for use in decision making based directly on the data, and it can certainly be argued that these might, in many cases, perform better than process representations if those representations are lacking in some aspect (see, for example, the hypothetico-inductive approach of [47,48]).

It is also well known that process representations are often sufficiently complex that they are underdetermined with respect to the data available to both define their correct functional form and, once a function form is defined, to identify the associated parameters and other auxiliary conditions (often including uncertain boundary conditions). The parameter values are often expected to be variable in both space and time, while modification of the auxiliary conditions can be used to compensate for various sources of error and uncertainty in the modelling process [49]. This has led to some philosophical discussion about the meaning of model testing and validation (e.g. [1,29,49–52]).

What is clear is that both the input variables for a model, and the observations with which we can check a model, are uncertain. Indeed, it is possible that some of the observations themselves might not be consistent with the physical principles that underlie the process representations in a model (for example, mass, energy and momentum conservation). It can be shown that not all the data collected, even with current measurement techniques, might be meaningful in this respect and could result in feeding *disinformation* into the model evaluation process (e.g. [28,53]).

One of the outcomes of this is that if we take such uncertainties into account in model evaluation, it reduces the potential for rejecting some models as useful hypotheses. Many models (both structures and parameter sets) might provide simulations that are (more or less) acceptable when evaluated against observed data. This is the *equifinality thesis* [54–59]. This is underdetermination in a sense that accepts the impossibility of converging on some optimal or ‘true’ model even given large datasets when there are significant epistemic uncertainties. The equifinality concept underlies the Generalized Likelihood Uncertainty Estimation (GLUE) methodology of Beven & Binley [60,61]. GLUE is a form of Bayesian reasoning in Bayes original sense of assessing models as hypotheses given some evidence. It is based on Monte Carlo model simulations, starting with some prior ensemble of model structures and parameter sets, but is generalized in the sense of allowing a range of updating methods and operators in evaluating each model realization. These can include formal multiplicative statistical likelihoods based on assumptions about the nature of the residuals, fuzzy measures and different fuzzy operators, binary limits of acceptability conditions or other user-defined criteria.

Recent applications of GLUE are based on trying to set some limits of acceptability based on what is known about uncertainty in the input and evaluation data. This allows for the rejection of models that do not satisfy those limits. In this way, it is less likely that models that will prove useful in prediction will be rejected just because of the epistemic errors in the data. That does not mean, of course, that all the models that might *not* be fit-for-purpose when used in prediction will be rejected, but in such cases it will be the case that rejection becomes more likely as more informative data, or different types of evaluation data, are collected and compared against model simulations.

We should note that this approach to testing models as hypotheses has been criticized because the rejection of models in this way does not have an axiomatic foundation, such as that which underlies probability theory (e.g. [46]). On the other hand, models that fall *within* the limits of acceptability can be evaluated with either a probabilistic or possibilistic (fuzzy) interpretation, and an axiomatic basis for the latter has been established by both Halpern [62] and Klir [63]. The question then is how to define limits of acceptability more rigorously as a form of a hypothesis test. Experience with this type of approach reveals that defining such limits of acceptability is difficult, particularly because of the potential for epistemic input errors that are not easily characterized or generated by stochastic processes, but which get processed through a complex nonlinear model construct before being compared with the evaluation data. The effect of an input error might then be non-stationary, conditional on the current state of the system and consequently rarely repeatedly sampled. That does, however, make the problem scientifically interesting and a driver for better measurement technologies.

## 5. Essentials of the problem of hypothesis testing

Let us take as a starting point an expectation that the data used to drive and evaluate models in the field sciences are epistemically uncertain in complex ways that are difficult to characterize (certainly using stochastic methods based on simple random processes will generally not represent the characteristics of uncertain variability well). This implies that model calibration and validation is a process of finding models that appear, at least, to be consistent with the uncertain data available. Different model structures and parameter sets within those model structures could be consistent in this sense. However, it can also be the case that all the models tried might prove to be inconsistent with the observations, even after allowing for the different sources of uncertainty (e.g. [64–67]; for cases where all the models tried have been rejected). Of course, if that is the case it should lead to questioning of both model definition and the meaning of the observations, which should hopefully lead to an advance in the science. We do not necessarily learn very much from accepting the best model currently available if it is not fit for purpose. We learn more from model rejection and consideration of the reasons for that rejection. More rigorous testing in this way might also lead to a more thoughtful approach to modelling [10]. That will not always happen, of course. The SWAT model, rejected in Hollaway *et al*. [67], will continue to be used widely around the world because it is freely available and comes with a database of parameter values.

The key phrase in that statement of the problem is finding models that appear to be consistent with the uncertain data available. This implies two requirements: firstly, an adequate method for carrying out the search in what will often be a high-dimensional space of both model structures and parameter sets (e.g. [59]) and secondly a method for defining what should be considered consistent. A limits of acceptability approach might be one possible way of doing so; a statistical likelihood would be another way where we are prepared to make strong aleatory assumptions about the nature of the model residuals. The latter approach can be difficult to justify in the environmental sciences because of the epistemic, often rather arbitrary, and sometimes disinformative nature of the uncertainties in both input and evaluation observations (e.g. [7,28]). Is there then some way of defining acceptability of a model, consistent with what we know about errors and uncertainties in the observations?

This will naturally depend on the nature of specific variables. Hydrological models, for example, are driven by estimates of precipitation and other meteorological variables over an area and are (mostly) evaluated only against estimates of stream discharge. The area involved might be a complete catchment area upstream of the discharge measurement site, or one of a number of spatial units or grid squares making up that catchment. In both cases, the required information is rarely measured directly, hence the reference to estimates (e.g. [68]). Precipitation and other meteorological variables are generally measured at a point rather than over an area (rainfall radars do give areal estimates but have their own uncertainty issues). Discharge is not generally measured directly on a continuous basis but is inferred from measurements of water levels at the catchment outlet, and a rating curve usually based on past discharge observations at points in time. Where there is no control structure, the observations used to derive the rating curve may themselves involve significant uncertainties depending on the method used, and are often limited at the lower and higher flows in which case low and high discharge estimates might depend on extrapolation of a fitted rating curve function. In such cases, different functional forms might result in quite different estimates of flood peaks and discharge time series (e.g. [69,70]). How to make such extrapolations is itself a form of epistemic uncertainty.

Similar issues arise for observables used for model evaluation in other environmental field sciences. Dealing with the observational uncertainties of evaluation variables is, however, potentially simpler than dealing with input uncertainties, because they can be considered as independent of the model being evaluated. Allowing for the impact of input uncertainties is more difficult because of the way that they will interact with the model structure to provide either acceptable or non-acceptable results. This will be the case for specific realizations of input error interacting with specific model configurations. This is evident, for example, in past studies that have attempted to evaluate the impact of input uncertainties in a hierarchical Bayes statistical framework. The BATEA (Bayesian Total Error Analysis) approach [71–73], for example, attempted to identify multipliers on the inputs for each rainfall event, drawn from an underlying random distribution. Effectively, these multipliers interacted with the model structure to provide better simulations, but resulted in a very wide distribution of potential multipliers when it came to make predictions for the first and subsequent events in prediction. The problem then is how to assess any impacts of input error independently of the model being evaluated.

This is a key issue, since how well a model can perform in evaluation (and the characteristics of the modelling residuals) will depend critically on the input data. Those input data (particularly when dealing with inputs over a catchment area in hydrological applications) will be subject to epistemic uncertainties that will be difficult to characterize and may vary in arbitrary (but not necessarily random ways) from event to event. At this point, the argument can be made that the information content of the available data should be the object of interest, rather than the uncertainty associated with that data (e.g. [74]). Following this line of reasoning has led to another form of testing models as hypotheses, based on simulation model performance relative to the best predictor that can extracted from the data alone (e.g. [75]). Relative performance in terms of information in this sense can be assessed in terms of entropy measures based on the probability distributions of the evaluation variable, model predictions and data-derived estimates. In this context, the requirement for an acceptable simulation model is that it should provide better predictions than purely data-derived estimates, i.e. that the modelling process is providing more information content.

This is an attractive idea but would appear to have some flaws in the case where there are important epistemic rather than aleatory uncertainties, such as where some periods of data are disinformative in the sense described earlier of being inconsistent with physical principles. That is not necessarily a problem for deriving estimates directly from the data. Data mining methods do not have to take account of physical constraints and might identify such cases simply as another class of behaviour (e.g. [76]). In addition, information content assessed by entropy measures does not take full account of the sequencing of observed and predicted values, only of the overall distribution. Thus, a simulation might get rejected by such a method, only because of epistemic uncertainties in the available data.

So is an alternative way of assessing the impact of epistemic input uncertainties possible? For this particular hydrological problem of predicting catchment discharges, there might be, at least for applications in some catchment areas. What we require, in terms of testing models as hypotheses, is an ensemble of model ensembles that are consistent with an assessment of uncertainty in the data used to force the model and the observations used to evaluate the model outputs. This can be done by specifying limits of acceptability for assessing the model predictions, where those limits take proper account of the limitations of the input and evaluation data.

## 6. Defining the impact of epistemic input errors on limits of acceptability

**Figure 1.**
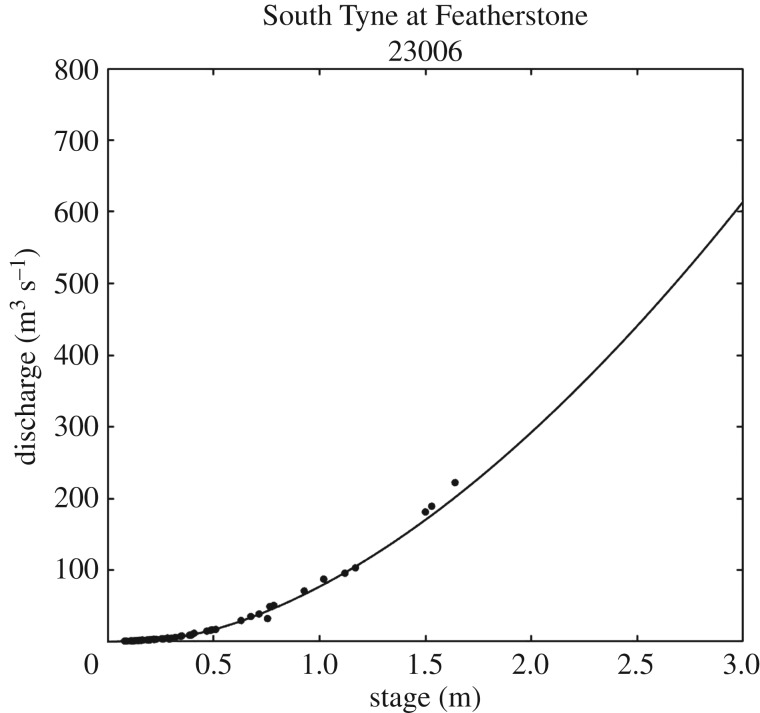
Stage-discharge rating curve for the South Tyne at Featherstone, showing the observations points and the function used by the Environment Agency to determine discharges at this site. Note that the maximum level recorded at this site is 2.75 m (well above the point measurements shown, but still within the capacity of the weir at the site).

To do so we can use an extension of the approach of Beven & Smith [28] where a method based on water mass balance constraints was used to identify rainfall-runoff events that should be considered as disinformative for model evaluation. We can use this information in a rather different way in testing models as hypotheses in a way that is independent of any particular model structure but is based only on the observational data alone and the principle of mass balance. The approach will be illustrated using the same dataset for the River South Tyne at Featherstone (UK Station 23006). This 332 km^2^ catchment has a well-maintained compound Crump weir, which was not overtopped during the period being considered, and remains modular throughout this range. It is, however, subject to some gravel accumulation that is removed intermittently (see https://nrfa.ceh.ac.uk/data/station/info/23006). The rating curve (figure 1) shows some deviation of check gaugings from the theoretical relationship towards higher discharges, but further measurements might reveal that this is more random observation error than a trend. The relationship is good, reflecting the weir construction, relative to many other gauging sites in the UK and elsewhere (e.g. [77]). Rainfalls over the catchment are estimated on the basis of five recording raingauges, all of which have periods of missing data. These stations are interpolated to estimate a continuous estimate of inputs to the catchment. This is not untypical of the type of dataset used in modelling in applying the science.

**Figure 2.**
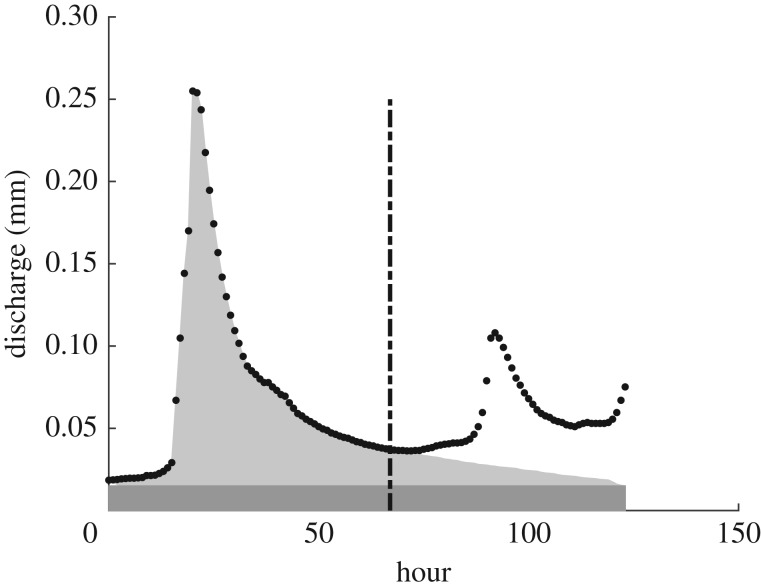
The use of a master recession curve to estimate the flow that would have occurred without the arrival of the new event. The total flow volume can then be used to calculate an effective runoff coefficient for that event.

**Figure 3.**
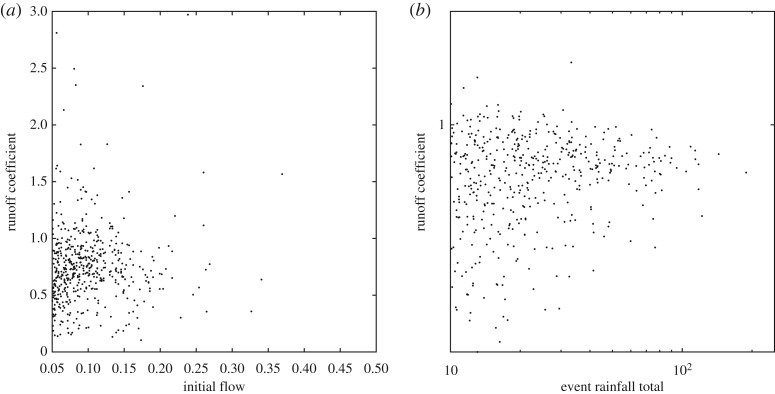
Plots of (*a*) event runoff coefficients against antecedent discharge and (*b*) event runoff coefficient against total storm rainfall for storm rainfall totals greater than 10 mm in the South Tyne at Featherstone.

The South Tyne catchment has a relatively fast and flashy response to rainfall. This is an advantage when trying to assess the characteristics of individual events, because it is easier to make an estimate of what the discharge from the catchment might have been if a new event had not occurred by extrapolating the recession curve of each event hydrograph using a master recession curve (see figure 2, [78] and [28] for details). This allows the mass balance for each event to be evaluated as a runoff coefficient (defined as the proportion of the rainfall appearing as discharge from the catchment). This type of separation of events was previously used in the context of unit hydrograph theory by Reed *et al*. [79], and was noted by Beven [35] as the only physically justifiable method of hydrograph separation. Beven & Smith [28] have already noted that despite the relatively good-quality data for this catchment application, event runoff coefficients can be sometimes more than 100% (i.e. more observed output than observed input) even for significant rainfall events, and also sometimes much lower than might be expected. The annual average runoff coefficient for this catchment, which integrates out the event to event variability, is 76%. The range of runoff coefficients from 761 events greater than 10 mm input in the period 1990–2003 are shown in figure 3.

In defining the limits of acceptability, we then need to allow for both the input and output uncertainties. In this case, the primary source of uncertainty is expected to be the epistemic uncertainty in the rainfall inputs but this might not always be the case (see for example, [68–70,77,80,81]; all of which give examples of significant uncertainties associated with discharge measurements). To allow for both sources of uncertainty in model evaluation, however, we can take advantage of the nature of the runoff coefficient in incorporating, at least implicitly, the effects of both input and output uncertainties (and any uncertainty in the recession curve extrapolation). The distribution of runoff coefficients over past events can then give an indication of the type of impacts of the epistemic input and output uncertainties that might be expected for events being used for model evaluation and for future events where model predictions might be required. In particular, we will examine that use of a conditional distribution of runoff coefficients over past events in defining limits of acceptability for model evaluation.

We would expect that the distribution of runoff coefficients will have some structure as well as some epistemic uncertainty. In particular, we expect runoff coefficients to increase with increasing rainfall inputs and also to increase with increasing antecedent wetness of a catchment. The effect of catchment wetness is shown is represented by the flow prior to the event which is generally accepted as a first-order index of catchment wetness. There may be other relevant variables, if there are observations available (spatial patterns of wetting and evapotranspiration can also play a role, but are much more difficult to estimate). These trends are shown in figure 3.

## 7. Use of runoff coefficient distributions in model evaluation

Consider first the case of model evaluation using data from a past period of hydrological observations. Where it is possible to use recession extrapolation to separate events we can derive a sample of runoff coefficients for past events directly from the observational data (as in figure 3). We note that it will be much more difficult to apply a mass balance constraint and derive event runoff coefficients in catchments with a large baseflow component that allows significant carry over of storage from event to event but is less of an issue for the wet, flashy catchment in this application.

When we then come to evaluate a model prediction for an event in the evaluation we have two sources of information. One is the value of the runoff coefficient for that event, calculated from the observed rainfalls and discharges, but for which we do not know the uncertainties (which might be significant) in either variable leading to that value. The other is the collection of runoff coefficients over all the events for which values can be calculated and, in particularly, for those events that are similar to the event under consideration. This gives an indication of the range of runoff coefficients that might potentially arise for similar events. Given a large enough sample of such events, we can estimate the potential range of runoff coefficients and use that to estimate potential outcomes, conditional on the calculated runoff coefficient for that event. This can be applied down to the time step level by using a ratio of runoff coefficients as follows:

7.1 $$F(Q|C_{i})=\frac{\sum_{1}^{J}w_{j}\{Q<Q_{t}C_{i}\text{/}C_{j}\}}{\sum_{1}^{J}w_{j}},$$

where *F*(*Q*|*C_i_*) is the cumulative function of estimated discharge *Q* at a given time step *t; Q_t_* is the observed discharge at time *t; C_i_* is the runoff coefficient for event *i* estimated from the observations for that event; *C_j_* is a sample from the distribution of runoff coefficients for *J* similar events; and *w_j_* is a membership weight associated with that *j*th sample*.* In this way, the distribution of potential discharges at any time step for that event can be calculated and, given that distribution, limits of acceptability for model runs decided. Note that in applying the storm runoff coefficients in this way there is no attempt to distinguish input uncertainty from discharge uncertainties. Indeed, this will generally be impossible when unknown input errors are processed through the catchment system in nonlinear ways. By looking at the potential uncertainties in observed responses from other similar events, however, we can have some estimate of the joint effect of both input and output uncertainties.

Two questions remain, however. First, how to decide on a sample of similar events from all the events available; and secondly how to decide on weights for the runoff coefficients for those similar events. There are a number of ways of resolving these issues. One is the approach of classifying events into groups on the basis of rainfall volume and antecedent discharge that was taken by Beven & Smith [28]. Within each class, the distribution of variability (in their case for model residuals) were all lumped together. Other possibilities might be to use a two-dimensional copula or weighted regression.

The approach taken here is to make use of the conditionality of the variability on a particular event that underlies equation (7.1) to choose an adequate local sample of similar events. Rather than using classes of events, this is achieved here by calculating a weighting function for all nearby events in the space of rainfall volumes and antecedent discharge (the approach could be extended to other relevant observables as appropriate). The issue then is what scaling to use in each dimension, when the hydrological effects of both discharge volume and antecedent discharge may operate nonlinearly on the runoff coefficients (figure 3). Here we are not really looking for covariation in any regression sense, but only a local sample of similar events, conditional on the observations for a chosen event. In this initial investigation, the Mahalanobis distance has been used to choose the 100 nearest neighbours, with the rainfall volume and antecedent flow axes scaled by the maximum values over all 761 events. The resulting weights could be interpreted either probabilistically or possibilistically; here we choose a possibilistic representation.

## 8. Limits of acceptability for evaluation events

Given the sample of runoff coefficients for the nearest-neighbour events, a distance weighted cumulative function of potential discharges can be formed. Here, a simple linear scaling of the Mahalabonis distance has been used, such that the weight would be one at zero distance and zero at the maximum distance over all the nearest neighbours. This can be interpreted as a form of fuzzy membership function for the set of nearby events. For an evaluation event, we know exactly where the calculated runoff coefficient for that event lies within the distribution of potential values as determined for the selected nearest neighbours, so the bounds on potential discharge from the catchment can be conditioned on that value. Since the runoff coefficient is an event characteristic, it can be applied as a simple multiplier for all the discharges during that event. This provides a very simple way of allowing for potential variability in the observed event responses arising from all sources of epistemic and aleatory error in both the inputs and outputs in terms of a proportional range.

**Figure 4.**
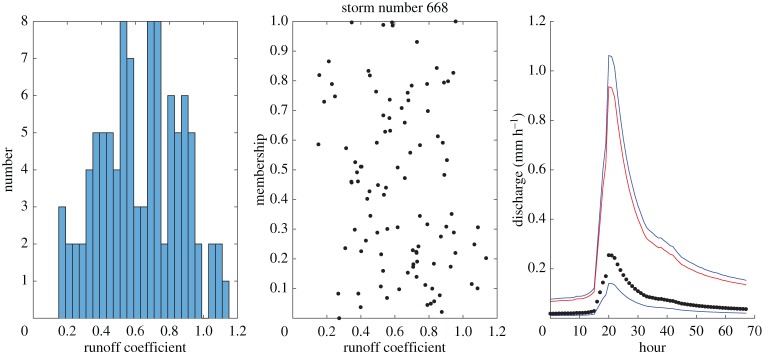
Event 668. Histogram of runoff coefficients from 100 nearest-neighbour events; empirical membership values of potential runoff coefficients from nearest-neighbour storms based on Mahalabonis distances; and derived limits of acceptability conditioned on runoff coefficient for the event (0.27; total rainfall 5.31 mm; initial flow 0.019 mm h^−1^). Blue upper and lower bound are determined from the range of the support events; red bound represents a runoff coefficient of 100% relative to the storm rainfall. (Online version in colour.)

**Figure 5.**
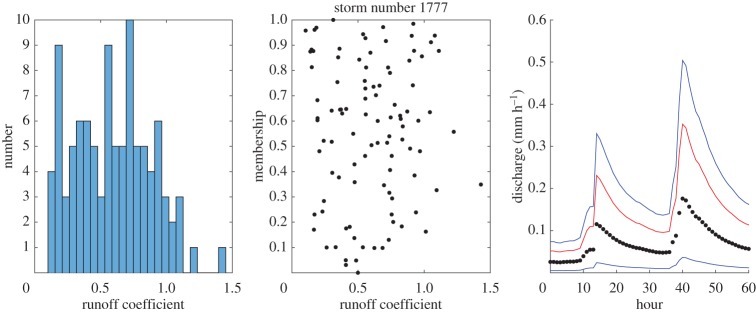
Event 1777. Histogram of runoff coefficients from 100 nearest-neighbour events; empirical membership values of potential runoff coefficients from nearest-neighbour storms based on Mahalabonis distances; and derived limits of acceptability conditioned on runoff coefficient for the event (0.45; total rainfall 10.89 mm; initial flow 0.023 mm h^−1^). Blue upper and lower bound are determined from the range of the support events; red bound represents a runoff coefficient of 100% relative to the storm rainfall. (Online version in colour.)

**Figure 6.**
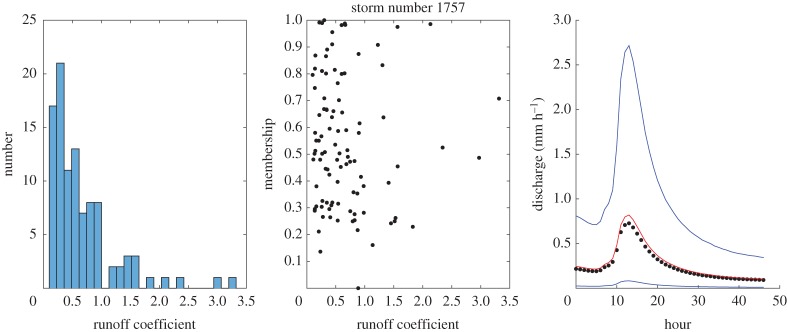
Event 1757. Histogram of runoff coefficients from 100 nearest-neighbour events; empirical membership values of potential runoff coefficients from nearest-neighbour storms based on Mahalabonis distances; and derived limits of acceptability conditioned on runoff coefficient for the event (0.89; total rainfall 6.41 mm; initial flow 0.217 mm h^−1^). Blue upper and lower bound are determined from the range of the support events; red bound represents a runoff coefficient of 100% relative to the storm rainfall. (Online version in colour.)

**Figure 7.**
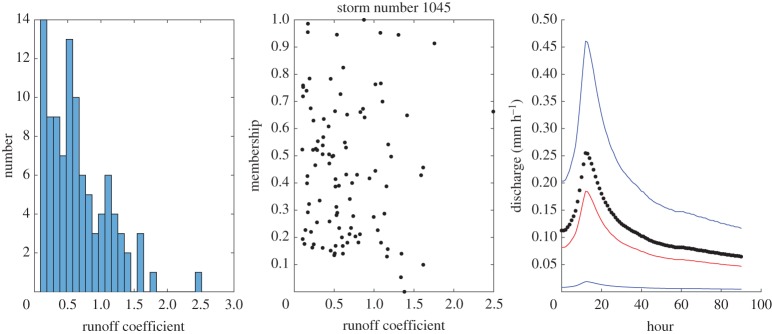
Event 1045. Histogram of runoff coefficients from 100 nearest-neighbour events; empirical membership values of potential runoff coefficients from nearest-neighbour storms based on Mahalabonis distances; and derived limits of acceptability conditioned on runoff coefficient for the event (1.38; total rainfall 6.28 mm; initial flow 0.11 mm h^−1^). Blue upper and lower bound are determined from the range of the support events; red bound represents a runoff coefficient of 100% relative to the storm rainfall. (Online version in colour.)

**Figure 8.**
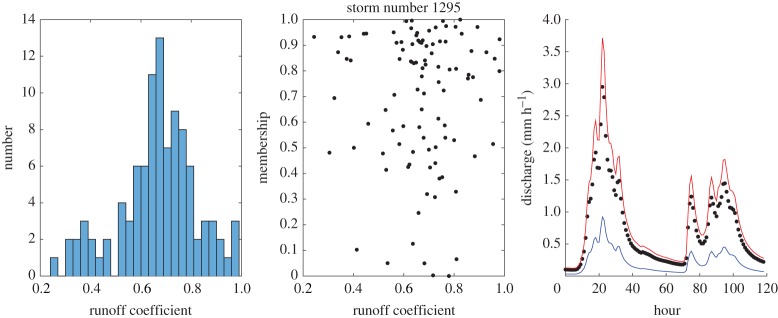
Event 1295 Histogram of runoff coefficients from 100 nearest-neighbour events; empirical membership values of potential runoff coefficients from nearest-neighbour storms based on Mahalabonis distances; and derived limits of acceptability conditioned on runoff coefficient for the event (0.7792; total rainfall 113.7 mm; initial flow 0.101 mm h^−1^). Blue upper and lower bound are determined from the range of the support events; red bound represents a runoff coefficient of 100% relative to the storm rainfall. (Online version in colour.)

Some example results for individual events are shown in figures 4–8 which show the histogram of runoff coefficients for all the nearest-neighbour events, the scaled membership values of potential runoff coefficients and the derived limits of acceptability based on the range of support of the nearest-neighbour events. The effect of conditioning on the calculated event runoff coefficient is most evident for the events that have either low (figure 4) or high (figure 7) values. Figures 6 and 7 show the effect of limiting the runoff coefficient to a value of 1 in model evaluation when we cannot expect a model that has a water balance constraint to produce runoff coefficients greater than 1. This is particularly the case in figure 7 when the observations exceed the unit bound because of the very high runoff coefficient calculated for this event. Figures 4 and 8 shows how the limits of acceptability are relatively narrower for events of either lower or higher initial flow and total inputs (as shown in the pattern of event runoff coefficients in figure 3).

It should be remembered that these limits of acceptability have been derived based only on the observed data and the principle of mass balance. No model runs have been made at this point, consistent with the principle expressed in Beven [56] that limits of acceptability should be derived independently of any model runs when used for testing models as hypotheses.

## 9. The potential for additional measurements to constrain epistemic uncertainties

Figures 4–8 show that the limits of acceptability for predicting discharges determined in this way can be rather wide. It must be remembered that this is because they represent the potential for variation in runoff coefficients over all ‘similar’ events based on the measures of total rainfall and initial flow as an index of the antecedent state of the catchment prior to an event. These measures might be refined; for example, the initial flow will be only a crude index of the spatial effects of patterns of storage and storage deficits resulting from evapotranspiration, particularly under summer conditions. But, these are not available as observations and in most cases might be less important than simply improving the estimates of rainfall inputs and discharge gauging in a catchment. In catchments subject to significant snow inputs, and where the discharge rating curve might be more uncertain than in the case of the South Tyne at Featherstone, then it might be that the limits of acceptability might be still wider.

It is, of course, possible to speculate that new sources of data might become available that could help in refining the model representations of a catchment. Spatial patterns can be observed by remote sensing, but at present the digital numbers of remote sensing images require interpretation by an (epistemically uncertain) model to derive estimates of hydrological variables of interest. The COSMOS method of soil moisture estimation [82–84] can integrate over an area that is as large as many model discretizations, but only in a way that varies in both depth and spatial extent with soil wetness. Other point measurements are sometimes available (for example, soil moisture or water table heights) but there both uncertainty and commensurability issues with such measurements when used for model evaluation (e.g. [7,85–87]).

## 10. Use of runoff coefficient distributions in prediction

The development of this way of allowing for input uncertainty in the hypothesis testing of hydrological models has been carried out in the practical context of the NERC funded Q-NFM project which is concerned with assessing the effectiveness of natural flood management (NFM) measures in mitigating flood peaks. NFM projects have received significant support from Government and Environment Agencies in recent years. The measures include afforestation on hillslopes and flood plains, small scale storages both in-stream and off-line, and re-instatement of meandering channels with the aim of ‘slowing the flow’. In many catchments, measures are being implemented without much in the way of evaluation or monitoring, in part because there are other co-benefits of carbon capture and the potential for increased biodiversity. Ideally, of course, the effectiveness of such schemes would be modelled before installation, at least for a range of demonstration or reference sites, but this requires simulations of change scenarios.

In making such prediction, we also expect that there will be errors associated with the input variables that are used to drive a model. In the GLUE methodology, predictions are made using an ensemble of ‘behavioural’ models that have survived the limits of acceptability evaluation process. The ensemble can be weighted according to how well a model in the behavioural set has performed in the evaluation process. If the limits of acceptability derived above were used in the model evaluation, then that survival will have reflected all the sources of uncertainty in the observational data but not necessarily in any new event that might need to be simulated.

For the case of NFM, the requirement is more to compare the responses under past observed flood events, with predictions of the same events with a range of potential implementations of NFM measures (e.g. [88,89]). In part, this is to test of whether those measures will have sufficient benefits to justify the investment (as assessed in terms of potential savings in flood damages) and in part to test whether there might be potential dis-benefits from such schemes. In larger catchments, slowing the flow in a downstream sub-catchment could actually lead to increases in the flood peak (known as the ‘synchronicity’ problem). For this type of problem, therefore, as well as the uncertainty in the behavioural model ensemble, there will be additional epistemic uncertainty associated with how to change the model configuration and parameters to explore the space of potential NFM measures. This is the subject of continuing research within the Q-NFM project.

## 11. Wider implications and conclusion

This study has illustrated how, in the inexact environmental sciences such as hydrology, epistemic uncertainties can lead to inconsistencies between observables and model principles, in this case the very basic hydrological principle of mass balance over a catchment area. In past work, we have suggested that this might lead to disinformation being fed into the model evaluation process with consequent potential for bias in model parameters and predictions [28]. Here an innovative methodology is suggested, of incorporating the potential variability in event mass balance for hydrological similar storm events directly into the model evaluation process through the use of limits of acceptability for hypothesis testing. In the example application, the dominant source of epistemic uncertainty is in the rainfall estimates over the catchment area, something that is extremely difficult to characterize using stochastic modelling, because of the limited number of gauges and expected non-stationarity in the error characteristics.

It is then shown that in this application the limits of acceptability are rather relaxed, particularly for relatively small events. The limits are, however, derived from the observations alone, and are realistic in reflecting the variability in responses for similar events. Having wide limits does not mean that they will not be useful in rejecting some models as hypotheses, even if a broad ensemble of models might be retained. Having realistic limits will also help avoid the possibility of rejecting models that might be useful for future predictions just because the model is being forced with poor data for some events. This then poses the question, of course, as to how we might be able to refine the model evaluation process. In this case, it is clear that the focus must be on improving the estimation of the inputs to the catchment. In other catchments, it might also be necessary to improve the estimates of stream discharges with which the model outputs will be compared. Finding new sources of data that can be used in evaluation, particularly variables that are commensurate with model variables other than the discharges that are normally used in model evaluation would also be useful.

The issues raised in this paper are not unique to hydrology and hydrological variables and models. They will be issues for other inexact sciences, particularly those for which boundary conditions are not easily controlled and replicate experiments are difficult or impossible. Most of the field or environmental sciences fall into this category. For those in which some fundamental principles of mass, energy, momentum or geochemical balances are applied in models of the system, then the type of methodology for model evaluation and hypothesis testing presented here might be a useful alternative to more traditional statistical methods based on model residuals.

## Data accessibility

The original data on which the figures in this paper is based are freely available from the Environment Agency under an OGL license.
